# Elevation shapes the seed endophytic bacteria richness and composition of *Taraxacum officinale*

**DOI:** 10.3934/microbiol.2026016

**Published:** 2026-06-24

**Authors:** Romy Moukarzel, Cristian-Andrei Costan, Philip E. Hulme

**Affiliations:** 1 Department of Pest-Management and Conservation, Lincoln University, Lincoln 7647, Canterbury, New Zealand; 2 Bioprotection Aotearoa, Lincoln University, Lincoln, Canterbury, New Zealand; 3 Foundation for Arable Research, Templeton, 7678, Canterbury, New Zealand

**Keywords:** seed microbiome, endophytic bacteria, ecological adaptability, plant-microbe interaction, plant invasion, weed, climate

## Abstract

*Taraxacum officinale*, a widely invasive plant species in New Zealand, thrives across environments, yet little is known about the seed endophytic microbial communities contributing to its adaptability. In this study, we characterized the bacterial community within *T. officinale* seeds across an elevation gradient of 10 to 720 meters above sea level. Using PCR-DGGE fingerprinting and 16S rRNA gene sequencing, we characterized bacterial community structures and assessed variations across sites. Bacterial richness declined significantly with increasing elevation, accompanied by distinct shifts in community composition. Non-metric multidimensional scaling revealed clear clustering of communities according to elevation, with higher elevation sites exhibiting more similar and less diverse microbiomes compared to lower elevation locations. A total of six dominant bacterial genera were identified: *Pseudomonas*, *Streptomyces*, *Clavibacter*, *Xanthomonas*, *Stenotrophomonas*, and *Erwinia*. These included core genera detected across sites and location-specific genera associated with particular elevations. These results suggested that elevation acts as an environmental filter shaping seed microbiome assembly, with potential implications for microbial transmission and plant adaptation. The functional consequences of these shifts for plant performance, adaptation, and invasion success remain unknown and require further investigation.

## 1. Introduction

Invasive alien plant species present significant ecological and economic challenges worldwide, impacting native biodiversity and ecosystem function [1]–[3]. One of the key factors contributing to the success of invasive alien plants is their ability to adapt and survive across a wide range of environments that has often been attributed to their inherent ecological plasticity or ability to rapidly adapt to local conditions [4],[5]. Less attention has been given to the role of microbial communities in shaping the plasticity and adaptation of alien plants, with most researchers focusing on soil-associated microbial communities [6],[7]. An underexplored aspect is how the seed microbiome might impact the adaptability of plant species to different environments. Seed-associated micro-organisms influence the metabolism, behavior, physiology, and development of their plant host, potentially enhancing the plant's ability to adapt to new environmental conditions [8]–[10]. Endophytes can improve seed viability and seedling vigor, improve germination and resilience to environmental stresses, and promote plant development [11]–[14]. Preliminary research suggests that the seed microbiome may be important in the performance of alien plants but the potential role in the establishment across a range of environments has not been explored [14],[15].

The composition of microbial communities in the seeds can be influenced by several factors, including host genotype [16]–[18] and the environment in which the plant is growing [19]–[22]. For invasive alien species, the microbial communities associated with the seeds may play an essential role in facilitating their establishment and spread in new ecosystems. It has been demonstrated that most bacterial and fungal endophytes isolated from seeds of *Anthemis cotula*, an invasive alien species in the Kashmir Himalaya, promotes plant growth, with some also exhibiting antagonistic effects against *Botrytis cinerea* when applied as a microbial consortium [14]. The removal of endophytes from invasive *Poa annua* seeds is associated with reduced seed germination, plant growth, and survivorship, suggesting that endophytes inherited from seeds may play a role in its invasive success in Antarctica [23]. Seed bacterial endophytes of invasive reed grass (*Phragmites australis*) are dominated by *Pseudomonas*, *Pantoea* and *Enterobacter* genera. Bacterial species from these genera have different functions, such as phosphorus solubilizing, protease production, anti-fungal and plant growth promotion, thus increasing the plant's competitive advantage in new environments [24].

*Taraxacum officinale*, commonly known as dandelion, is a perennial weed native to Europe and Asia but has become naturalized across the globe, particularly in temperate regions [25]. It thrives in various environments, including lawns, roadsides, meadows, and disturbed soils [26]. Its adaptability has made *T. officinale* one of the most successful invasive plants worldwide [27], frequently categorized as a noxious weed in many regions [28]. Seeds are the primary means by which *T. officinale* reproduces and a single plant can produce thousands of seeds annually, with each seed head capable of producing between 150 and 200 seeds [25]. Reproduction occurs primarily through dispersal of cypselae (achenes), which consist of the seed enclosed within maternal fruit tissues [29]. Additionally, dandelions exhibit sexual reproduction (via seeds) and asexual reproduction through apomixis, where seeds can develop without fertilization, producing genetically identical offspring [30]. This reproductive flexibility enables *T. officinale* to spread effectively and maintain stable populations, even in isolated or disturbed environments [31].

Despite considerable evidence of the phenotypic plasticity of *T. officinale* and its ability to adapt to many environments [27],[32],[33], there have been no studies to explore whether the seed microbiome may play a role. Having this information could provide an understanding of the successful invasion of dandelion in environments. In this work, we hypothesized that if bacterial communities were a function of environmental drivers, then a) bacterial communities would cluster within locations across an environmental gradient as a result of adaptation to local conditions, and b) similarity among bacterial communities would be a function of elevation. We then explored the composition of these communities to determine if they contain bacterial groups that might facilitate plant performance.

## 2. Materials and methods

### 2.1. Sampling sites and seed collection

*T. officinale* seeds were collected from seven distinct geographical locations along an elevation gradient across the Canterbury region of New Zealand (Table 1). In each location, seeds were collected from five different populations, with a 15 to 20-meter radius separating each population to ensure spatial independence. The term “seed” is used throughout the manuscript to refer to the *Taraxacum* cypsela (achene). The sampling took place at the end of autumn, extending from late May to early June (2024). After collection, seeds from each population were stored separately in zip lock bags for later analysis. The sampling sites were selected along an elevational gradient ranging from 10 to 720 meters above sea level, with elevation serving as a proxy for environmental variation because it reflects changes in climatic, edaphic, and vegetation-related factors across the region.

**Table 1.** Geographical location and elevation information of the collected *Taraxacum officinale* seeds.

Nb	GPS coordinates	Geographical location	Habitat	Elevation (m)
1	43°13′55.5″S 171°43′25.3″E	Castle Hill	Roadsides	720
2	43°20′16.5″S 171°55′52.5″E	Springfield	Park	390
3	43°23′19.7″S 172°01′14.2″E	Sheffield	Park	300
4	43°28′14.1″S 172°05′33.9″E	Darfield	Park	200
5	43°32′35.1″S 172°09′20.0″E	Charing Cross	Roadsides	147
6	43°30′40.8″S 172°22′16.7″E	Rolleston	Roadsides	50
7	43°38′21.8″S 172°29′04.8″E	Lincoln	Roadsides	10

### 2.2. Surface sterilization of seeds

A single *T. officinale* seed of each of the five collected populations from each site were washed with tap water and air dried in a laminar flow hood. Each seed was soaked in 96% ethanol for 15 seconds, followed by immersion in a 2.5% sodium hypochlorite solution for 2 min. The seeds were then washed three times in sterile water for 1 minute each to remove the sterilizing agents. After surface sterilization, each seed was cut into small pieces using a sterilized blade and stored in a 1.5 mL Eppendorf tube (Axygen, USA) at −80 °C for DNA extraction.

### 2.3. DNA extraction

Each seed was soaked in 300 µL sterile Millipore water, and 1 µL of 20 mM propidium monoazide (PMA) (Biotium, USA) was added to each tube to prevent DNA amplification from surface microbes killed by the sterilization method [34]. Following PMA treatment, the seeds were stored at −80 °C for 2 hrs. Five sterilized steel beads (4 mm) (BioEcho Life Sciences, Germany) and 300 µL Qiagen lysis buffer were added to each tube before being shaken for 15 min on a Vortex-Genie 2 (Mo Bio laboratories, Inc., SA) to ensure that seed fragments were macerated. Total DNA was extracted using the DNeasy PowerPlant Pro Kit (Qiagen Laboratories, Hilden, Germany) following manufacturer instructions with a final elution volume of 50 µL. DNA quality and concentration were measured by a Micro-volume Spectrophotometer (Thermo Fisher Scientific NanoDrop Lite) and gel electrophoresis. DNA yield ranged from approximately 15–20 ng per seed and was comparable across samples from the geographical locations.

### 2.4. PCR amplification

PCR reactions were conducted in 25 µL volumes containing 18.75 µL ultra-pure water (Life Technologies, Thermo Fisher Scientific, USA), 2.5 µL of 10× PCR buffer with 20 mM MgCl_2_ (2 mM final MgCl_2_), 1 µL of 10 µM from each of the forward and reverse primers, 1.25 U FastStart Taq DNA Polymerase (0.25 µL) (Roche diagnostics, Mannheim, Germany), and 1 µL (~15–20 ng) DNA template. The bacterial 16S rRNA gene V3 region was amplified using primers 357 GC (39 bp GC clamp-CCT ACG GGA GGC AGC AG) and 518 R (ATT ACC GCG GCT GCT GG), which are commonly employed for DGGE-based analysis of bacterial community composition. PCR thermal cycles were run as follows: Initial denaturation at 95 °C for 3 min, 35 cycles at 94 °C for 60 s, 55 °C for 60 s, and 72 °C for 60 s, followed by a final extension period at 72 °C for 7 min. The PCR products were verified by electrophoresis on 1.5% agarose gels containing 2 µL RedSafe™ nucleic acid staining solution (20,000×; iNtRON biotechnology, UK) in 1× TAE buffer at 90 V for 40 min. The determination of band sizes was facilitated using a 1 kb plus DNA ladder (New England Biolab), and the bands were visualized using a Gel Doc Go Imaging System (Bio-Rad, USA). All samples produced clear PCR amplicons and were therefore considered suitable for subsequent DGGE analysis.

### 2.5. Denaturing gradient gel electrophoresis

Denaturing gradient gel electrophoresis was performed using the Cipher DGGE Electrophoresis System (C.B.S. Scientific, USA). Eight µL of PCR product was mixed with 3 µL of 2× loading dye and loaded onto an 8% (w/v) polyacrylamide gel with 45%–65% linear denaturing gradient for total bacteria. The gels were run in 0.5× TAE buffer at 58 °C and 90 V for 16 hrs. After electrophoresis, the gels were washed with reverse osmosis (RO) water to remove the remaining TAE buffer, fixed in 250 mL of 1× fixation solution for 10 min, and stained with silver staining solution for 10 min. The stain was removed, and the gels were washed with RO water for 2 min. The gels were then developed in developer solution for 40 min, fixed again in 250 mL of 1× fixation solution for 5 min, photographed, and analyzed for bacterial communities. The recipes for the solutions and reagents used are provided in the supplementary information.

### 2.6. Band excisions and sequencing

**Figure 1.**
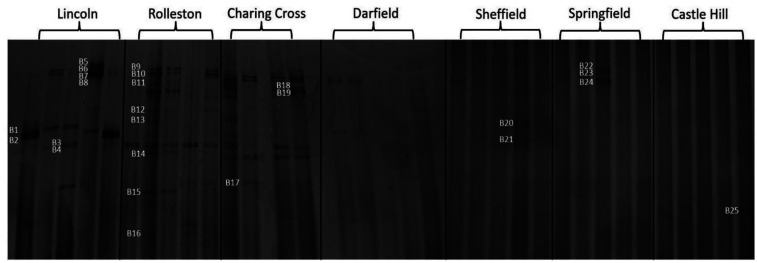
Denaturing gradient gel electrophoresis of seed endophytic bacteria of *Taraxacum officinale* (5 populations) in each of the seven different geographical locations. The DGGE band numbers (B1–B25) represent bacterial taxa that are excised and sequenced.

The most representative and distinctive DGGE bands from each geographical location were excised, labelled, and placed into 2 mL tubes. Each tube received 50 µL of nucleic-acid-free water and was stored overnight at 4 °C. Following storage, the tubes were heated at 60 °C for 30 min and centrifuged at 16,000 g for 20 min. The supernatant was transferred to a new 0.7 mL tube, avoiding the acrylamide gel pieces [35]. An aliquot (1 µL) of the supernatant was used as the template for re-amplification with the same primers and PCR conditions as previously described. The PCR products were purified using a PCR clean-up kit (Ultraclean, Carlsbad, CA) before being sequenced at the Lincoln University sequencing facility using Sanger dideoxy sequencing technology (Applied Biosystems, HITACHI, 3500 xL, Genetic Analyzer, New Zealand). Sequences were edited using BioEdit software (Hall, 1999) and compared by BLAST to those in the NCBI database to identify similar sequences. The total number of bands excised and sequenced was 25 bands (Figure 1). The sequences of the DGGE bands were submitted to the NCBI database under the accession numbers PQ584727–PQ584742.

### 2.7. Statistical analysis

To examine bacterial richness within each location, the number of DGGE bands per lane was used as an indicator of bacterial taxonomic richness. The effect of elevation on bacterial richness was analyzed using a general linear model (GLM) in IBM SPSS Statistics 26 [36] (George and Mallery, 2019). Pairwise comparisons of bacterial richness between elevation gradients were conducted using the least significant difference (LSD) test.

To investigate whether bacterial communities in *T. officinale* seeds are structured by the elevation gradient, DGGE gel bands from each site were analyzed using Phoretix 1D Pro 16.2 software (BioSystematica) to generate community matrix data. The binary matrix indicates the presence (1) or absence (0) of each band detected in all the lanes. A single band was considered one bacterial taxon. Resemblance matrices for bacterial community profiles were constructed by calculating the similarities between each pair of samples using the Jaccard similarity index as implemented in Primer 7 (PRIMER-E Ltd., Plymouth Marine Laboratory, UK).

Main and pairwise permutational multivariate analysis of variance (PERMANOVA) tests were conducted to assess significant differences (p < 0.05) in bacterial community composition across elevation gradients. Non-metric multidimensional scaling (nMDS) plots with average bootstrapping were generated in Primer 7 to visualize clustering patterns in bacterial community distribution across locations and to evaluate whether similarity among communities correlated with elevation.

## 3. Results

### 3.1. Bacterial taxa richness across elevation gradients

**Figure 2.**
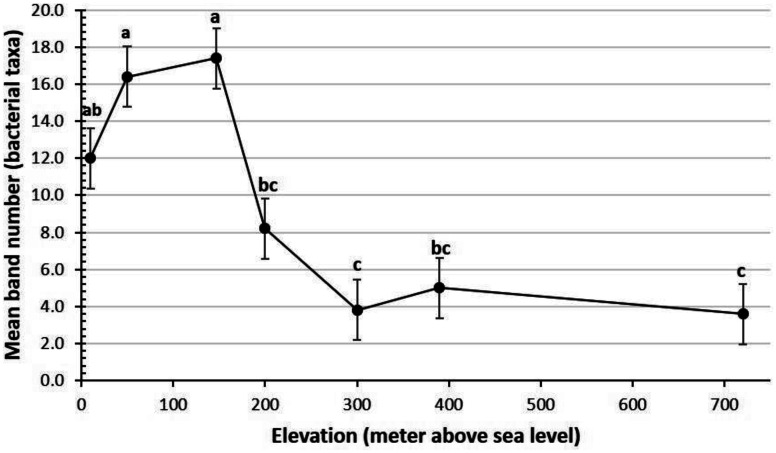
Mean band number representing bacterial taxa diversity in each elevation gradient.

The mean band number representing bacterial taxa in *T. officinale* seeds varied significantly across elevations (p < 0.001). Locations at lower elevations such as Lincoln (10 m asl), Rolleston (50 m asl), and Charing Cross (147 m asl) exhibited the highest bacterial richness (Figure 2). A significant reduction in bacterial richness was observed at Darfield (200 m asl), with continued to decline in richness with increased elevations in locations, including Sheffield (300 m asl), Springfield (390 m asl), and Castle Hill (720 m asl) (Figure 2).

### 3.2. Elevation gradients influence seed endophytic bacterial communities

**Figure 3.**
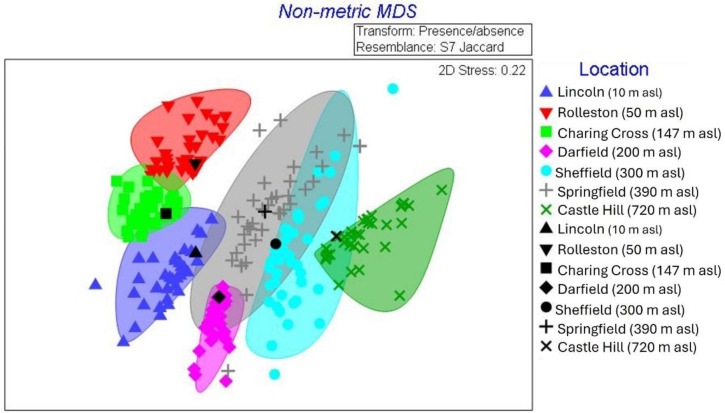
Non-metric multidimensional scaling (MDS) plot based on Jaccard similarity matrices generated from presence-absence data of bacterial community profiles associated with *Taraxacum officinale* seeds in seven locations along an altitudinal gradient. (▲) Lincoln, (▼) Rolleston, (■) Charing Cross, (♦) Darfield, (●) Sheffield, (+) Springfield, and (x) Castle Hill. Each colored symbol represents an individual seed bacterial community profile, while the corresponding black symbols indicate the centroid (group average) for each location. Shaped ellipses represent the dispersion of samples within each location. Communities that are more similar are ordinated closer together. Stress = 0.22.

Elevational gradients had a significant impact on the seed endophytic bacterial communities of *T. officinale* (PERMANOVA, p = 0.001, Table S1). Total bacterial communities at lower elevations (Rolleston and Charing Cross) differed significantly from those at elevations above 200 m asl (Figure 3, Table S2). Additionally, Lincoln (10 m asl) exhibited a distinct bacterial profile compared with Rolleston, Charing Cross, and Castle Hill (Figure 3, Table S2). Endophytic bacterial communities at higher elevations (Sheffield, Springfield, and Castle Hill) showed greater similarity to one another (Table S2). It was also observed that Springfield and Sheffield had more variable bacterial communities, while Darfield had a more consistent bacterial composition within this location, which was reflected by the spread and compactness of clusters (Figure 3).

### 3.3. Seed endophytic bacterial community composition

The representative and distinctive DGGE bands (n = 25) from the different locations were excised, re-amplified, and sequenced. In all, 17 (68%) of the sequenced DGGE bands were identified as bacteria, while the remainder produced multiple sequence signals (MSS) that were unreadable. Most sequences were assigned to *Pseudomonas* sp. (1–4), *Streptomyces* sp. (1–3), and *Stenotrophomonas* sp. (1–2) followed by *Clavibacter* sp., *Erwinia* sp., and *Xanthomonas* sp. (Table 2). *Pseudomonas* sp. 3 and *Streptomyces* sp. 3 were present at all locations while *Streptomyces* sp. 2 and *Pseudomonas* sp. 2 were detected only at locations at lower elevations (Lincoln, Rolleston and Charing Cross). *Xanthomonas* sp. was detected in Rolleston, Charing Cross, and Darfield. *Clavibacter* sp., *Pseudomonas* sp. 4, *Stenotrophomonas* sp. (1–2), and *Streptomyces* sp. (1–2) were shared among multiple locations. The following species were each unique to a single location: *Pseudomonas* sp.1 at Lincoln, an uncultured bacterium at Rolleston, and *Erwinia* sp. at Castle Hill (Table 2).

**Table 2.** Sequenced bands excised from DGGE gel of bacterial communities associated with *Taraxacum officinale* seeds at each geographical location with their highest identity matches.

Band #	Geographical location	Base pair	Bacterial taxa closest match	Similarity %	Accession #
B9	Present in all the geographical locations	180	*Pseudomonas* sp. 3	99%	NR117820.1
B10	Present in all the geographical locations	180	*Pseudomonas* sp. 3	99%	NR117820.1
B24	Present in all the geographical locations	157	*Streptomyces* sp. 3	98%	NR115403.1
B22	Lincoln, Rolleston, Charing Cross, Springfield, Castle Hill	174	*Pseudomonas* sp. 4	98%	NR170438.1
B23	Lincoln, Rolleston, Charing Cross, Springfield, Castle Hill	180	*Pseudomonas* sp. 4	98%	NR170438.1
B1	Lincoln, Rolleston, Charing Cross, Darfield	155	*Streptomyces* sp. 1	97%	NR180531.1
B2	Lincoln, Rolleston, Charing Cross, Darfield	NA	MSS*	NA	NA
B3	Lincoln, Rolleston, Charing Cross, Darfield	NA	MSS	NA	NA
B21	Lincoln, Rolleston, Charing Cross, Sheffield	170	*Clavibacter* sp.	97%	NR115038.2
B4	Lincoln, Rolleston, Charing Cross	160	*Streptomyces* sp. 2	97%	NR117992.2
B6	Lincoln, Rolleston, Charing Cross	NA	MSS	NA	NA
B7	Lincoln, Rolleston, Charing Cross	175	*Pseudomonas* sp. 2	97%	NR169460.1
B8	Lincoln, Rolleston, Charing Cross	NA	MSS	NA	NA
B11	Lincoln, Rolleston, Charing Cross	NA	MSS	NA	NA
B12	Lincoln, Rolleston, Darfield	160	*Stenotrophomonas* sp. 1	90%	NR121739.1
B17	Lincoln, Rolleston, Charing Cross	NA	MSS	NA	NA
B18	Rolleston, Charing Cross, Darfield	175	*Xanthomonas* sp.	97%	NR136457.1
B20	Rolleston, Charing Cross, Sheffield	174	*Clavibacter* sp.	97%	NR115038.2
B14	Rolleston, Charing Cross	175	*Stenotrophomonas* sp. 2	97%	NR117406.1
B15	Rolleston, Charing Cross	NA	MSS	NA	NA
B16	Rolleston, Charing Cross	176	*Stenotrophomonas* sp. 2	97%	NR117406.1
B19	Rolleston, Charing Cross	NA	MSS	NA	NA
B5	Lincoln	170	*Pseudomonas* sp. 1	98%	NR136473.1
B13	Rolleston	175	Uncultured bacterium	97%	KY642335.1
B25	Castle Hill	183	Erwinia sp.	97%	NR118854.1

*MSS = multiple sequence signal

### 3.4. Seed bacterial genera distribution across elevation gradients

**Figure 4.**
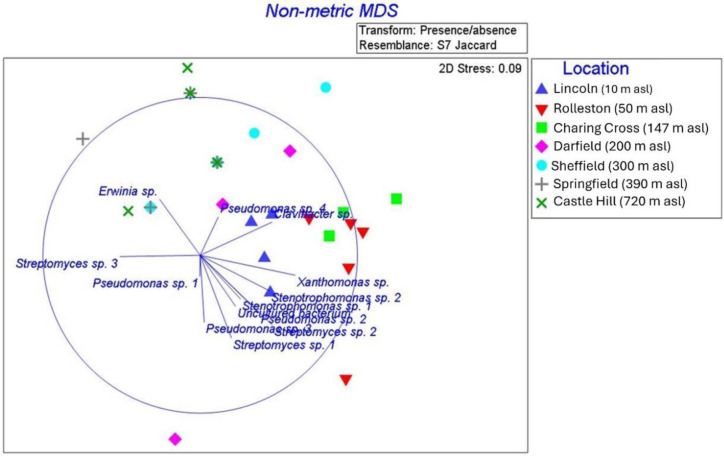
Non-metric multidimensional scaling (MDS) plot showing the distribution of bacterial genera (vectors) associated with *Taraxacum officinale* seeds across seven different geographical locations. (▲) Lincoln, (▼) Rolleston, (■) Charing Cross, (♦) Darfield, (●) Sheffield, (+) Springfield, and (x) Castle Hill. Points represent Objects (geographical location). Objects that are more similar to one another are ordinated closer together. The axes and orientation of the plot are arbitrary.

The bacterial communities associated with *T. officinale* seeds differed across the elevation gradient (PERMANOVA, p < 0.001). Sites at lower elevations (Lincoln, Rolleston, Charing Cross, and Darfield) presented a cluster with similar seed bacterial composition across these sites. In contrast, seeds collected from higher elevations (Sheffield, Springfield, and Castle Hill) harbored distinct bacterial communities, as reflected by their separation from the main cluster in the nMDS plot. Specifically, Castle Hill was characterized by a strong association with *Erwinia* sp. and *Streptomyces* sp. 3, as indicated by the directional vectors pointing toward Castle Hill in the nMDS plot (Figure 4). In the lower elevation cluster, taxa such as *Pseudomonas* sp. 4, *Xanthomonas* sp., and *Clavibacter* sp. were more prevalent, particularly in Lincoln, Rolleston, and Charing Cross. Moreover, shorter vectors for *Pseudomonas* sp. 2, Streptomyces sp. 1, and *Stenotrophomonas* sp. 1 were more evenly distributed across the elevation gradient (Figure 4).

## 4. Discussion

We are the first to characterize the endophytic bacterial community composition associated with the seeds of *T. officinale*. Six bacterial genera, including *Pseudomonas* spp., *Streptomyces* spp., *Clavibacter* spp., *Xanthomonas* spp., *Stenotrophomonas* spp., and *Erwinia* spp. were identified based on the 16S rRNA Sequencing of representative DGGE bands in each geographical location. These bacterial genera were also isolated from the seeds of other invasive alien species such as *Lactuca serriola* [37] and *Phragimates australis* [24]. In this study, denaturing gradient gel electrophoresis (DGGE) was used to study the microbial communities associated with *T. officinale* seeds from different geographical locations. DGGE is a molecular fingerprinting technique that separates PCR-amplified DNA fragments based on their sequence-dependent denaturation properties, enabling assessment of microbial community composition and detection of diversity shifts among samples [38],[39]. This method is particularly useful for comparing microbial communities across environmental gradients and identifying broad community-level patterns. Although DGGE has lower taxonomic resolution than newer sequencing approaches and may underestimate the complexity of highly diverse microbial communities [40], the patterns observed in this study were robust and consistent across elevation gradients. While higher-resolution approaches such as next-generation sequencing (NGS) would likely provide greater granularity [41],[42] and potentially identify a larger number of microbial clusters at individual locations, they would be unlikely to substantially alter the overall interpretation of elevation-driven shifts in bacterial community composition.

This study demonstrates that elevation is a strong environmental filter shaping the richness and composition of seed endophytic bacterial communities in *T. officinale*. A decline in bacterial taxa richness with increasing elevation was observed, alongside distinct shifts in community structure. Although DGGE was used in this study, similar elevation-associated patterns in plant-associated microbial communities have been reported using high-resolution sequencing approaches in other plant tissues and species [43],[44], suggesting that these ecological trends are robust regardless of the molecular method employed. This is consistent with seed microbiome studies showing that seed-associated microbial communities are shaped by ecological filtering, host selection, geography, and environmental conditions, and that seeds can act as important vectors for microbial transmission between plant generations [12],[14],[24],[45],[46]. More specifically, studies have shown that seed microbial communities can vary among environments and along elevation-related gradients, supporting the idea that local conditions influence seed microbiome assembly [47],[48]. These findings also align with broader microbial biogeography studies showing that elevation affects microbial diversity through changes in temperature, moisture, UV radiation, soil properties, and plant physiology [49]. Although these environmental variables were not measured directly in this study, research in the Canterbury high country has demonstrated strong relationships between elevation, soil development, vegetation history, and soil physicochemical characteristics [50],[51]. For example, shifts in vegetation communities and soil-forming processes across elevational gradients can alter nutrient availability, organic matter accumulation, and microbial habitat quality, thereby influencing the diversity and composition of plant-associated microorganisms [52]. Consequently, the distinct bacterial communities observed among elevation bands may reflect environmental filtering driven by a combination of climatic, edaphic, and vegetation-related factors rather than elevation alone.

The higher bacterial richness observed at lower elevations is consistent with studies showing that microbial diversity generally decreases with increasing elevation due to harsher environmental conditions and reduced resource availability [43],[53]. Warmer temperatures and greater plant productivity at lower elevations likely promote more diverse microbial recruitment and transmission into seeds. In contrast, higher elevations impose abiotic stressors such as lower temperatures, increased UV exposure, and shorter growing seasons, which can limit microbial colonization and survival [54]. These constraints likely contribute to the reduced richness and increased similarity of seed bacterial communities observed at higher elevations in this study.

The significant differences in bacterial community composition across elevations further indicate that elevation influences not only diversity but also the selection of specific microbial taxa. The clustering of communities from higher elevation sites suggests stronger environmental filtering, resulting in more conserved and less diverse microbiomes. Similar patterns have been reported in soil and plant-associated microbial communities, where environmental stress leads to convergence in community composition [49]. The greater variability observed at intermediate elevations (e.g., Sheffield and Springfield) may reflect transitional zones where environmental filtering and ecological drift influence microbial assembly [55].

The dominance of genera such as *Pseudomonas*, *Streptomyces*, and *Stenotrophomonas* across sites highlights the importance of these genera as core members of the seed microbiome. The consistent presence of bacterial genera such as *Pseudomonas* and *Streptomyces* across all elevations suggests that these genera may represent a conserved core microbiome that is vertically transmitted or selectively maintained due to functional importance. Core seed microbiomes are increasingly recognized as important contributors to early plant development and resilience [45]. In contrast, some bacterial groups were detected only at lower elevations, indicating that certain members of the seed microbiome may be more sensitive to environmental conditions and potentially acquired horizontally from local environments. The presence of *Erwinia* sp. at the highest elevation (Castle Hill) is notable, as members of this genus are often associated with plant pathogenicity but can also exist as endophytes under certain conditions [52]. This may reflect altered plant–microbe interactions under stress conditions, where opportunistic genera become more prevalent.

The observed differences in bacterial genera distribution across elevations further suggest that seed microbiomes are shaped by environmental filtering and host-mediated selection. The association of *Pseudomonas*, *Xanthomonas*, and *Clavibacter* with lower elevation sites may reflect greater microbial availability and plant productivity, whereas the dominance of *Streptomyces* and *Erwinia* at higher elevations may indicate selection for stress-tolerant or competitive genera. These findings align with the concept that plant-associated microbiomes are assembled through a combination of environmental filtering, dispersal limitation, and host selection [56].

We are the first to examine the effect of elevation on endophytic bacterial diversity within *T. officinale* seeds, revealing a decline in bacterial diversity and shifts in community composition at higher elevation sites (Sheffield, Springfield, and Castle Hill). While other researchers have not explored seed endophyte richness across elevation gradients, similar patterns have been reported in soil microbiome studies [57]–[60]. By extending this concept to the seed microbiome, our findings of this study demonstrate that seed endophytic bacterial communities vary along environmental gradients, highlighting the potential for elevation-driven environmental factors to influence microbial transmission to the next plant generation. This is particularly important given that most researchers have focused on soil and root-associated microbiomes, with limited attention to seeds, despite their critical role in plant establishment, stress tolerance, and invasion success [12],[14].

## 5. Conclusion

This study provides novel evidence that elevation is a key environmental driver shaping seed endophytic bacterial communities in *T. officinale*. A decline in bacterial richness and a shift in community composition was observed with increasing elevation, indicating strong environmental filtering of the seed microbiome. The consistent presence of genera such as *Pseudomonas* and *Streptomyces* across sites suggests the existence of a conserved seed microbiome, while the occurrence of location-specific genera highlights the influence of local environmental conditions on microbial assembly. These findings extend current understanding by demonstrating that seed-associated microbiomes, like those of soil and roots, vary systematically along environmental gradients and may play a role in plant adaptation to contrasting environments. Given the importance of seed microbiota in early plant establishment and stress tolerance, these results suggest that elevation-driven shifts in microbial communities could contribute to the ecological success and broad distribution of *T. officinale*. In future studies, researchers should investigate how variations in seed morphological and physiological traits, such as seed size, mass, and germination success, relate to shifts in seed-associated microbial communities across environmental gradients. Studies should also examine the functional roles and transmission of these endophytes across generations, as well as their interactions with fungal communities, to better understand how the plant microbiome responds to environmental gradients.

## Use of AI tools declaration

The authors declare they have not used Artificial Intelligence (AI) tools in the creation of this article.


