# Supplementary material for: Elevation shapes the seed endophytic bacteria richness and composition of Taraxacum officinale
Source: AIMS Microbiol. 2026 Jun 24;12(2):377–92. doi: 10.3934/microbiol.2026016 (PMC13370273; doi:10.3934/microbiol.2026016)
Supplement: Supplementary file 1 [file microbiol-12-02-016-s001.pdf]

AIMS Microbiology

AIMS Microbiology, 12(2): 377–392.
DOI: 10.3934/microbiol.2026016
Received: 11 May 2026
Revised: 08 June 2026
Accepted: 18 June 2026
Published: 24 June 2026

https://www.aimspress.com/journal/microbiology

Research article

Elevation shapes the seed endophytic bacteria richness and composition of *Taraxacum officinale*

Romy Moukarzel[1,*], Cristian-Andrei Costan[3] and Philip E. Hulme[1,2]

[1] Department of Pest-Management and Conservation, Lincoln University, Lincoln 7647, Canterbury, New Zealand
[2] Bioprotection Aotearoa, Lincoln University, Lincoln, Canterbury, New Zealand
[3] Foundation for Arable Research, Templeton, 7678, Canterbury, New Zealand

* **Correspondence:** Email: romy.moukarzel@lincoln.ac.nz.

Supplementary

1. Recipes of reagents used for DGGE

a) 2× DGGE gel loading dye (10 mL):
- 2% bromophenol blue: 0.25 mL 0.05%
- 2% xylene cyanol: 0.25 mL 0.05%
- 100% glycerol: 7.0 mL 70%
- Millipore water: 2.5 mL

b) Denaturing polyacrylamide (PA):
0% and 100% denaturing polyacrylamide (PA) 8% used for total fungi and total bacteria.
0% denaturing PA* 8% (per 100 mL)
- 40% Acrylamide:Bisacrylamide (37.5:1) (Bio-Rad, USA): 20 mL
- 50× TAE: 1 mL
- 100% glycerol: 2 mL
- Millipore water: to 100 mL

100% denaturing PA* 8% (per 100 mL)
- 40% Acrylamide:Bisacrylamide (37.5:1) (Bio-Rad, USA): 20 mL
- Urea (Sigma-Aldrich, USA): 42 g
- Formamide (Sigma-Aldrich, USA): 40 mL
- 50× TAE: 1 mL
- 100% glycerol: 2 mL
- Millipore water: to 100 mL

*Store in the dark at room temperature, low heat (≤ 37 °C) to dissolve.

c) 50× TAE (1 L)
- Tris Base: 242 g
- Millipore water: 500 mL
- Glacial acetic acid: 57.1 mL
- 0.5 M EDTA (pH 8): 100 mL
- Millipore water: to 1000 mL

d) 8× fixative solution (1 L)
- 96% ethanol 800 mL
- Acetic acid 40 mL
- Millipore water 160 mL

e) 1× fixative solution (2 L)
- 8× fixative solution: 250 mL
- Millipore water: to 2000 mL

f) Silver stain (500 mL; for 2 gels, to prepare fresh just before staining)
- 1× fixative solution: 500 mL
- Silver nitrate: 1 g

g) Developer (500 mL for 2 gels)
- 3% NaOH: 250 mL
- Millipore water: 250 mL
- Formaldehyde: 1 mL

Table S1. main effect of geographic location on bacterial composition.

Bacteria						
Factors						
Name	Abbrev.	Type	Levels			
Location	Lo	Fixed	7			
PERMANOVA table of results						
Unique						
Source	df	SS	MS	Pseudo-F	P (perm)	perms
Lo	6	35120	5853.3	1.6051	0.001	998
Res	28	1.0211E+05	3646.6			
Total	34	1.3723E+05				

Table S2. Pairwise comparison of the bacterial communities between different locations.

Groups	t	P (perm)	Unique perms
Lincoln, Rolleston	1.2368	0.044	125
Lincoln, Charring Cross	1.2959	0.013	126
Lincoln, Darfield	1.1929	0.077	126
Lincoln, Sheffield	1.1168	0.087	126
Lincoln, Springfield	1.1905	0.097	125
Lincoln, Castle Hill	1.41	0.008	126
Rolleston, Charring Cross	1.3223	0.024	126
Rolleston, Darfield	1.5959	0.011	126
Rolleston, Sheffield	1.2808	0.019	125
Rolleston, Springfield	1.2443	0.045	126
Rolleston, Castle Hill	1.4942	0.009	125
Charring Cross, Darfield	1.4161	0.018	126
Charring Cross, Sheffield	1.3219	0.008	126
Charring Cross, Springfield	1.3311	0.005	126
Charring Cross, Castle Hill	1.5349	0.007	126
Darfield, Sheffield	1.1778	0.072	106
Darfield, Springfield	1.1994	0.101	126
Darfield, Castle Hill	1.488	0.006	126
Sheffield, Springfield	0.85083	0.866	103
Sheffield, Castle Hill	0.97458	0.477	111
Springfield, Castle Hill	1.0101	0.358	94

AIMS Press

© 2026 the Author(s), licensee AIMS Press. This is an open access article distributed under the terms of the Creative Commons Attribution License (https://creativecommons.org/licenses/by/4.0)
